# Supplementary material for: Targeting the TCA cycle can ameliorate widespread axonal energy deficiency in neuroinflammatory lesions
Source: Nat Metab. 2023 Jul 10;5(8):1364–81. doi: 10.1038/s42255-023-00838-3 (PMC10447243; doi:10.1038/s42255-023-00838-3)
Supplement: Supplementary file 1 — Reporting Summary [file 42255_2023_838_MOESM1_ESM.pdf]

nature portfolio

Corresponding author(s): Thomas Misgeld/ Martin Kerschensteiner

Last updated by author(s): May 22, 2023

Reporting Summary

Nature Portfolio wishes to improve the reproducibility of the work that we publish. This form provides structure for consistency and transparency in reporting. For further information on Nature Portfolio policies, see our Editorial Policies and the Editorial Policy Checklist.

Statistics

For all statistical analyses, confirm that the following items are present in the figure legend, table legend, main text, or Methods section.

n/a	Confirmed	
☐	☒	The exact sample size (*n*) for each experimental group/condition, given as a discrete number and unit of measurement
☐	☒	A statement on whether measurements were taken from distinct samples or whether the same sample was measured repeatedly
☐	☒	The statistical test(s) used AND whether they are one- or two-sided<br>*Only common tests should be described solely by name; describe more complex techniques in the Methods section.*
☐	☒	A description of all covariates tested
☐	☒	A description of any assumptions or corrections, such as tests of normality and adjustment for multiple comparisons
☐	☒	A full description of the statistical parameters including central tendency (e.g. means) or other basic estimates (e.g. regression coefficient) AND variation (e.g. standard deviation) or associated estimates of uncertainty (e.g. confidence intervals)
☐	☒	For null hypothesis testing, the test statistic (e.g. *F*, *t*, *r*) with confidence intervals, effect sizes, degrees of freedom and *P* value noted<br>*Give P values as exact values whenever suitable.*
☒	☐	For Bayesian analysis, information on the choice of priors and Markov chain Monte Carlo settings
☒	☐	For hierarchical and complex designs, identification of the appropriate level for tests and full reporting of outcomes
☒	☐	Estimates of effect sizes (e.g. Cohen's *d*, Pearson's *r*), indicating how they were calculated

Our web collection on statistics for biologists contains articles on many of the points above.

Software and code

Policy information about availability of computer code

Data collection	Olympus MPE-RS, Olympus FV1200 MPE and Olympus FV1000 confocal microscopy system were used for in vivo imaging.<br>Olympus FV10-ASW (version 4.2) confocal microscopy system and Leica SP8 confocal microscopy system were used for in situ experiments.<br>Zeiss Imager Z1 Apotome 2 microscope was used for COX histochemsitry analysis.<br>3DHistech Pannoramic P250 II whole slide scanner was used for human sections.<br>Thermo Fisher Scientific Q-Exactive HF mass spectrometer coupled with Easy nLC-1200 nano UHPLC was used for mass spectrometry.
Data analysis	Open source software: ImageJ/Fiji (version 1.51u) for processing of images; GraphPad (version 7) for statistics and figure representation; Microsoft Excel 2022 for statistics; MaxQuant (Version 1.6.3.3 or 1.6.10.43) for processing of mass spectrometry; Reactome (version 7.4) for proteomics pathways analysis; R studio (version 4.2.2) and Python (version 3) package nezzworker for proteomics data processing; Adobe Photoshop CS6 for image processing; Adobe Illustrator CS6 for figure representation; SlideViewer software (v2.3, 3DHistech), Definiens Developer XD (version 2.7) and Visiopharm (version 2021.09) for human image analysis; QSM™ data analysis platform for metabolic modelling; Biorender for illustration display.

For manuscripts utilizing custom algorithms or software that are central to the research but not yet described in published literature, software must be made available to editors and reviewers. We strongly encourage code deposition in a community repository (e.g. GitHub). See the Nature Portfolio guidelines for submitting code & software for further information.

Data

Policy information about availability of data

All manuscripts must include a data availability statement. This statement should provide the following information, where applicable:

- Accession codes, unique identifiers, or web links for publicly available datasets
- A description of any restrictions on data availability
- For clinical datasets or third party data, please ensure that the statement adheres to our policy

All proteomic dataset generated within this study are deposited online to the ProteomeXchange Consortium via the PRIDE partner repository with the dataset identifier PXD032363 and at github (https://github.com/engelsdaniel/mitoproteomics). The codes used to reanalyze the single-cell data from Schattling et al. and the halflives data from Fornasiero et al. are publicly available on the github (https://github.com/engelsdaniel/mitoproteomics). Source data files for Figures and Extended Data Figures are provided with this paper. All additional data that support the findings of this study are available upon reasonable request to the corresponding authors. The transgenic mouse lines (Thy1-mitoTwitch2b and Thy1-PercevalHR) and the plasmids (pAAV.hSyn.Idh3a.p2a.tdTomato, pAAV.hSyn.Mdh2.p2a.tdTomato, pAAV.hSyn.Cre.p2a.tdTomato, pAAV.hSyn.Ateam1.03 and pAAV.hSyn.SypHer3s) are available upon request.

Research involving human participants, their data, or biological material

Policy information about studies with human participants or human data. See also policy information about sex, gender (identity/presentation), and sexual orientation and race, ethnicity and racism.

Reporting on sex and gender	5 female and 2 male patients
Reporting on race, ethnicity, or other socially relevant groupings	N/A
Population characteristics	25-77 years of age<br>Diagnostics: multiple sclerosis
Recruitment	Samples were composed of tissues derived from two primary sources: autopsies and biopsies, primarily carried out in a diagnostic context, without pro-active recruitment of patients for this study, and samples were gathered based on the patient's prior consent or appropriate legal permission to use tissues for research purposes. Together this makes a self-selection bias less likely to affect the observed results.
Ethics oversight	The Use of human samples was in accordance with institutional ethical guidelines and approved by the ethics committee of the University of Geneva (Switzerland).

Note that full information on the approval of the study protocol must also be provided in the manuscript.

Field-specific reporting

Please select the one below that is the best fit for your research. If you are not sure, read the appropriate sections before making your selection.

☒ Life sciences ☐ Behavioural & social sciences ☐ Ecological, evolutionary & environmental sciences

For a reference copy of the document with all sections, see nature.com/documents/nr-reporting-summary-flat.pdf

Life sciences study design

All studies must disclose on these points even when the disclosure is negative.

Sample size	Although no power calculation was performed prior to study design to predetermine the sample sizes, the number of mice used ensured statistically relevant results are comparable to those reported in previous publications using similar experimental design (see Ref: Nikic et al., Nature Medicine 2011; Sorbara et al., Neuron 2014; Witte et al., Neuron 2019; Fecher et al., Nature Neuroscince 2019)<br>All number of mice and cells are reported in the respective sections (i.e. Figure Legends, Online methods and source data files).
Data exclusions	Pre-established exclusion criteria were used in this study- animals showing signs of traumatic damage after laminectomy were excluded from the analysis based on the animal protocol. Mice or tissue showing insufficient labeling or staining, or structures showing insufficient signal to noise ratios were not analyzed; no other data points were excluded.
Replication	All animal experiments in this study include at least three biological replicates. The number of replicates is mentioned for each experiment in the figure legend. All attempts at replication were successful, except when the data exclusion criteria were met prior to analysis.
Randomization	Female and male were equally allocated into control and experimental groups if not explicitly mentioned otherwise (sample collection for mass spectrometry/ proteomics analysis in particular, only male mice were used). The experiments were not powered for independent analysis of male and female mice, but separate analysis of female and male mice did not reveal any major sex-specific effects in our analyses of ATP/ADP levels.

Blinding

Data collection and analysis was performed blind for figure 4 and figure 6 to avoid the selection bias.
Others were not applied due to the nature of the binary type of experiment (obvious disease phenotype: control vs EAE). All analysis were performed using images displayed on a gray scale to avoid color-driven analysis bias.

Reporting for specific materials, systems and methods

We require information from authors about some types of materials, experimental systems and methods used in many studies. Here, indicate whether each material, system or method listed is relevant to your study. If you are not sure if a list item applies to your research, read the appropriate section before selecting a response.

Materials & experimental systems

n/a	Involved in the study
☐	☒ Antibodies
☐	☒ Eukaryotic cell lines
☒	☐ Palaeontology and archaeology
☐	☒ Animals and other organisms
☒	☐ Clinical data
☒	☐ Dual use research of concern
☒	☐ Plants

Methods

n/a	Involved in the study
☒	☐ ChIP-seq
☒	☐ Flow cytometry
☒	☐ MRI-based neuroimaging

Antibodies

Antibodies used

or tissue staining:
Primary antibodies:
Idh3a, Novus Biologicals, NBP1-32396; Dilution: IF - 1: 400
Idh2, Thermo Scientific, 702713; Dilution: IF - 1: 400
Mdh2, Novus Biologicals, NBP1-32259; Dilution: IF - 1 : 400
RFP, Novus Biologicals, NBP1-97371 ; Dilution: IF - 1: 1000
GFP, Abcam, ab13970; Dilution: IF - 1: 1000
NeuN, clone A60, Sigma, MAB377; Dilution: IF - 1: 400
Secondary antibodies:
Anti-chicken IgY-Alexa Fluor 488, Thermo Scientific, A-11039; Dilution: IF - 1: 1000
Anti-rabbit IgG-Alexa Fluor 488, Thermo Scientific, A32731; Dilution: IF - 1 : 1000
Anti-mouse IgG-Alexa Fluor 594, Thermo Scientific , A-21125; Dilution: IF - 1: 1000
Anti-rabbit IgG-Alexa Fluor 594, Thermo Scientific, A-11012; Dilution: IF - 1: 1000
For mitochondrial isolation:
Mouse IgG1-APC, Miltenyi Biotec, 130-113-758

Validation

All antibodies used in this study were commercially available and validated by the manufacturers or used in previous studies:
Idh3a, Novus Biologicals, NBP1-32396 : Data sheet
Idh2, Thermo Scientific, 702713 : RRID:AB_2734812; Data sheet
Mdh2, Novus Biologicals, NBP1-32259 : Data sheet
(The staining of Idh3a, Idh2 and Mdh2 were performed on tissues from Thy1-mitoRFP mice, where analysis can be restricted to red fluorescent protein (RFP)-tagged neuronal mitochondria.)
RFP, Novus Biologicals, NBP1-97371 : Data sheet; 5 citations (PMID: 30159312/PMID: 30158698)
GFP, Abcam, ab13970 : Data sheet (ab13970 staining GFP in GFP-transfected NIH/3T3cells.); 2965 citations
NeuN, Sigma, MAB377 : Data sheet; 4999 citations (PMID: 31100147/PMID: 31474560)
Anti-chicken IgY-Alexa Fluor 488, Thermo Scientific , A-11039 : RRID:AB_2534096; 168 citations
Anti-rabbit IgG-Alexa Fluor 488, Thermo Scientific, A32731 : RRID:AB_2633280; 414 citations
Anti-mouse IgG1-Alexa Fluor 594, Thermo Scientific, A-21125 : RRID:AB_2535767; 22 citations
Anti-rabbit IgG-Alexa Fluor 594, Thermo Scientific , A-11012 : RRID:AB_2534079; 215 citations
Mouse IgG1-APC, Miltenyi Biotec, 130-113-758 : RRID:AB_2733439; Ref: doi: 10.1016/j.ab.2009.02.040

Eukaryotic cell lines

Policy information about cell lines and Sex and Gender in Research

Cell line source(s)	HEK293T, ATCC, crl-3216.
Authentication	None of the cell lines used were authenticated.
Mycoplasma contamination	We confirm that all cell lines were tested negative for mycoplasma contamination.
Commonly misidentified lines (See ICLAC register)	N/A

Animals and other research organisms

Policy information about studies involving animals; ARRIVE guidelines recommended for reporting animal research, and Sex and Gender in Research

Laboratory animals	All experiments were performed on either postnatal day 3 pups or adult (age from 2 to 6 months) mice according to the protocols on a C57BL/6 (strain designation C57BL/6J, Jackson Laboratories) background, crossbred in our animal facilities.<br>The following transgenic animals were used:<br>Thy1-mitoGrx-roGFP x Thy1-OFP mice<br>Thy1-PercevalHR<br>Thy1-mitoTwitch2b x Thy1-OFP<br>Rosa26-MitoTag<br>Thy1-mitoRFP mice<br>Thy1-OFP
Wild animals	Study did not involve any wild animals.
Reporting on sex	Both sexes were used and separate analysis of female and male mice did not reveal any major sex-specific effects (in our analyses of ATP/ADP levels). Exclusively, only male mice were used in the mass spectrometry/ proteomics analysis.
Field-collected samples	No field-collected animals were used in the study.
Ethics oversight	All animal experiments were performed in accordance with regulations of the relevant animal welfare acts and protocols approved by the local animal ethics committee of the state of Bavaria (Regierung von Oberbayern) in accordance with European guidelines.

Note that full information on the approval of the study protocol must also be provided in the manuscript.
